# The Perceived Effectiveness of Secure Messaging for Medication Reconciliation During Transitions of Care: Semistructured Interviews With Patients

**DOI:** 10.2196/36652

**Published:** 2022-08-03

**Authors:** Julianne E Brady, Amy M Linsky, Steven R Simon, Kate Yeksigian, Amy Rubin, Alan J Zillich, Alissa L Russ-Jara

**Affiliations:** 1 Center for Healthcare Organization and Implementation Research VA Boston Healthcare System Boston, MA United States; 2 General Internal Medicine VA Boston Healthcare System Boston, MA United States; 3 General Internal Medicine Boston University School of Medicine Boston, MA United States; 4 Center for the Study of Healthcare Innovation, Implementation & Policy VA Greater Los Angeles Healthcare System Los Angeles, CA United States; 5 Department of Medicine David Geffen School of Medicine University of California Los Angeles Los Angeles, CA United States; 6 Department of Psychiatry Boston University School of Medicine Boston, MA United States; 7 Department of Pharmacy Practice College of Pharmacy Purdue University West Lafayette, IN United States; 8 Center for Health Information and Communication US Department of Veterans Affairs Veterans Health Administration, Health Services Research and Development CIN 13-416 Indianapolis, IN United States; 9 Regenstrief Center for Healthcare Engineering Purdue University West Lafayette, IN United States

**Keywords:** medication reconciliation, patient portals, telemedicine, pharmacist-patient relationship, medication errors

## Abstract

**Background:**

Medication discrepancies can lead to adverse drug events and patient harm. Medication reconciliation is a process intended to reduce medication discrepancies. We developed a Secure Messaging for Medication Reconciliation Tool (SMMRT), integrated into a web-based patient portal, to identify and reconcile medication discrepancies during transitions from hospital to home.

**Objective:**

We aimed to characterize patients’ perceptions of the ease of use and effectiveness of SMMRT.

**Methods:**

We recruited 20 participants for semistructured interviews from a sample of patients who had participated in a randomized controlled trial of SMMRT. Interview transcripts were transcribed and then qualitatively analyzed to identify emergent themes.

**Results:**

Although most patients found SMMRT easy to view at home, many patients struggled to return SMMRT through secure messaging to clinicians due to technology-related barriers. Patients who did use SMMRT indicated that it was time-saving and liked that they could review it at their own pace and in the comfort of their own home. Patients reported SMMRT was effective at clarifying issues related to medication directions or dosages and that SMMRT helped remove medications erroneously listed as active in the patient’s electronic health record.

**Conclusions:**

Patients viewed SMMRT utilization as a positive experience and endorsed future use of the tool. Veterans reported SMMRT is an effective tool to aid patients with medication reconciliation. Adoption of SMMRT into regular clinical practice could reduce medication discrepancies while increasing accessibility for patients to help manage their medications.

**Trial Registration:**

ClinicalTrials.gov NCT02482025; https://clinicaltrials.gov/ct2/show/NCT02482025

## Introduction

Medication discrepancies are associated with unintended consequences for patients, including adverse drug events (ADEs), rehospitalizations, and emergency department visits [1-3]. Medication discrepancies, defined as unintended differences between documentation in a patient’s medical record and what the patient reports taking [4], commonly include omissions, commissions, and incorrect dose or frequency. Identifying medication discrepancies during transitions from hospital to home—a time of increased risk for discrepancies—can benefit patients and save costs via decreased rehospitalizations and less emergency department utilization [3].

Nearly 60% of patient records contain at least one medication discrepancy [5]; therefore, identifying discrepancies is a crucial step to reduce ADEs. Medication reconciliation is a process by which the medications that a patient reports taking are compared with the medications listed in their health record with subsequent resolution of any identified discrepancies. The final step of the medication reconciliation process involves communicating the corrected list to the patient, caregivers, and clinical teams [6]. Medication reconciliation *prior* to hospital discharge is known to decrease patient readmissions and emergency department visits [7]. However, less is known about effective and efficient medication reconciliation processes that occur during the care transition *after* hospital discharge.

There has been substantial advancement in the integration of information technology into the electronic health record (EHR) to identify and resolve medication discrepancies during hospital admissions [8,9] and in the outpatient setting [1,9-12]; however, little research has focused on health information technologies to identify and address medication discrepancies during patient transitions between care settings.

One potentially useful technology to address medication discrepancies in the postdischarge period is a web-based patient portal. These portals are integrated into a health system’s EHR and allow patients to have greater access to and control of their health information. Common features include the ability to request prescription refills, manage appointments, and send secure electronic messages (ie, secure messaging) with health-related questions to their health care clinicians [13-15]. The development and advancement of secure messaging within patient-facing platforms has allowed for greater communication between health care clinicians and patients [16,17]. Secure messaging within patient portals may allow for improved health outcomes [18,19]. Several tools now leverage secure messaging to address health concerns, such as diabetes, hypertension, and weight management [20,21].

Previously, our group developed and tested the Secure Messaging for Medication Reconciliation (SMMRT) tool [22] as a solution for patients to use secure messaging asynchronously to help identify medication discrepancies after being discharged from an inpatient setting to home. We conducted a formal usability evaluation of SMMRT with patients in a human-computer interaction laboratory [23]. In this study, our objective was to characterize how patients perceived the ease of use and effectiveness of the SMMRT intervention after using SMMRT in a real-world setting. We sought to identify features of SMMRT that patients perceived as most and least effective and to assess how this tool could be improved for patients in the future.

## Methods

### Trial Setting, Participants, and Intervention

This research is part of a larger study [23,24] that included a randomized controlled trial of SMMRT [25], which was conducted at 1 tertiary Veterans Affairs (VA) Medical Center to analyze the effectiveness of asynchronous, patient portal–based communication via secure messaging, between patients and clinicians for medication reconciliation. Briefly, the trial recruited patients from acute hospital settings or subacute rehabilitation centers who were prescribed 3 or more medications, were being discharged home (as opposed to a rehabilitation facility), passed the Callahan Six-Item Screener for cognitive impairment [26], and had a home computer and internet access. Patients randomly assigned to the intervention group were asked to use SMMRT for medication reconciliation once they returned home; control patients received usual care [25].

### SMMRT Trial Intervention

**Figure 1:**
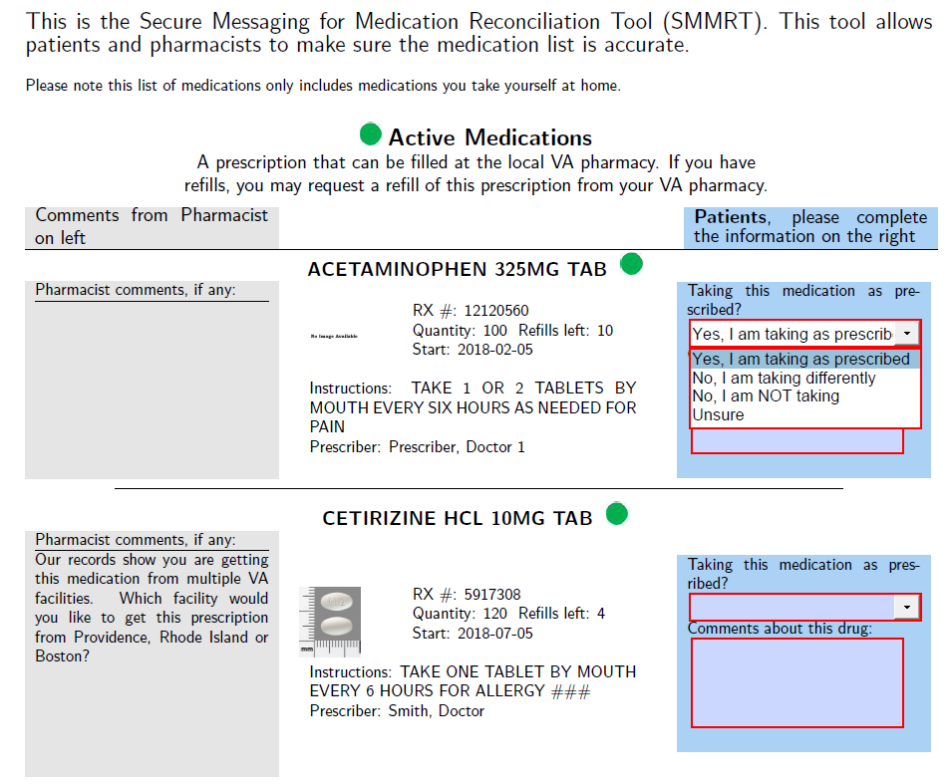
Example of the Secure Messaging for Medication Reconciliation Tool (SMMRT).

SMMRT is an interactive PDF that allows for a patient and clinician to conduct medication reconciliation after hospital discharge using secure messaging. Each SMMRT form contains the medication names, dosages, directions, and images of all active, expired, and pending medications documented in the patient’s EHR. Patients can review their medication list and select from options in a dropdown menu to indicate whether they are taking the medication as directed, as shown in Figure 1.

Within 3 business days of hospital discharge, all patients enrolled in the intervention arm of the trial were sent a SMMRT form to review. Prior to hospital discharge, research assistants (RAs) trained patients on how to use the patient portal and SMMRT by helping the participants log into their patient portal account and allowing patients to use a sample SMMRT for practice. This training was intended to prepare patients so they could use SMMRT later at their home. Technical support was available to patients after discharge via the study’s contact. Patients were instructed to use SMMRT to review their medications on their own and return it to the study’s clinical pharmacists via secure messaging within 10 days of receipt. If a patient did not return SMMRT within 10 days, one of the study’s clinical pharmacists contacted the patient via telephone and talked with the patient to complete SMMRT together. This allowed patients to view SMMRT at home while discussing their medications with the clinical pharmacist. Once SMMRT was complete, either by the patient via secure messaging or by the clinical pharmacist completing SMMRT with the patient over the phone, the clinical pharmacist reviewed SMMRT information and updated the EHR documentation (eg, by removing or adding medications to the patients’ records) to reconcile any discrepancies. Analyses of medication reconciliation accuracy was outside the scope of qualitative interviews, which were focused on patients’ perceptions.

### Study Participants, Recruitment, and Procedures

Participants who were enrolled in the intervention arm from April 2019 to September 2019 were eligible to participate in an interview, regardless of SMMRT completion status. We contacted potential participants sequentially, according to the date of their study enrollment, and invited them to participate via a mailed invitation with up to 2 follow-up phone calls until we reached our goal of 20 patient interviews. Due to project time constraints, sequential sampling was conducted. A total of 29 participants were invited to participate, with 8 nonrespondents, 1 participant declining to participate with no response given, and 1 participant who became ineligible since he was readmitted to the hospital. Participants were involved in 30-minute semistructured phone interviews; they received US $50 for their time if they completed any portion of the interview.

Semistructured interviews were conducted between July 2019 and September 2019, within 2 months of the patient’s participation in the SMMRT trial intervention arm. All interviews with participants were conducted via phone by 1 of 2 RAs, both with master’s degrees and prior experience conducting qualitative interviews (JEB, KY). No new themes were identified after 10 participants, indicating adequate data saturation [27]. To minimize personal bias, the primary RA who conducted the patient interviews and individuals who served as qualitative analysts were not involved in the initial development of SMMRT, patient recruitment for the clinical trial, or patient training on SMMRT. Interviews were audio-recorded with the permission of the participant and transcribed verbatim.

### Ethical Approval

All study procedures and documents were approved by the VA Boston Healthcare System Institutional Review Board on 21 May, 2018 (protocol number: IRB#3156).

### Interview Content

The primary objective of interviews was to characterize patients’ perceptions of the ease of use and effectiveness (ie, ability to identify and reconcile medication discrepancies) of SMMRT. We also explored perceptions of the VA patient portal, MyHealtheVet, as it related to the use of secure messaging and completion of medication reconciliation. Interview questions (Multimedia Appendix 1) were developed under the guidance of 2 research physicians (AML and SRS), a research psychologist (AR), and a PhD scientist with extensive experience in qualitative methods (ALRJ). Questions were developed to probe patients about their experiences using SMMRT and MyHealtheVet and to discuss features of SMMRT that emerged as most effective and those features that patients felt were unnecessary or counterproductive. We also sought to understand why patients did not complete or return SMMRT. The interview guide was pilot tested within the research team and refined for clarity.

### Analysis

We followed a qualitative analysis approach described by Bradley et al [28] for health services research. We did not have any preconceived themes prior to analysis; rather, interview transcripts were analyzed using an inductive, qualitative analysis approach to identify emergent themes [28]. To begin, 2 transcripts were randomly selected, read, and analyzed independently by 2 members of the qualitative team (ALRJ, JEB). They discussed potential themes until reaching consensus on an initial list [28]. The analysts then independently re-analyzed the same initial 2 transcripts using the initial list of themes (Table 1) and then again reviewed and revised themes and discussed coding discrepancies until consensus agreement was reached [28]. The remaining 18 transcripts were then coded by JEB, who discussed any potential newly identified themes or coding difficulties with ALRJ until consensus was reached to minimize personal bias [28]. To ensure quality, a total of 5 (25%) transcripts were independently analyzed and discussed by these 2 analysts on an iterative, periodic basis over the course of the data analyses [28]. Coding of all transcripts was documented using NVivo qualitative analysis software [29]. Frequencies and proportions of responses were calculated based on interviewee responses and relevant baseline data collected as part of the larger trial. This manuscript was prepared based upon the Standards for Reporting Qualitative Research (SRQR) [30].

**Table 1:** Key themes used for coding, along with example quotes from patients.

Theme	Definition	Example quote(s)
Barriers to use of asynchronous communication platform	Barriers the participant faced while using components of MyHealtheVet (MHV) software that were also needed for SMMRT^a^ form study process. This can include difficulties related to logging into MHV, the use of the secure messaging platform, downloading SMMRT, uploading SMMRT, and internet connection issues. Exclusions: This does not include barriers of difficulties relating to using SMMRT from pdf once downloaded.	“I did have a problem [with MyHealtheVet] because they had to [do] something so I could send the messages...”
Ease of use of asynchronous communication platform	Ease of use of using the MHV platform in the context of the SMMRT study. This can include comments about ease of use of logging into MHV, the use of the secure messaging platform, downloading SMMRT, and attaching SMMRT to the secure message. Exclusion: This does not include comments about using SMMRT once downloaded.	“I couldn’t remember my login or my password, so I had to keep going back and getting a new one.”
Barriers to using SMMRT	Difficulties the patient experienced while using SMMRT PDF once downloaded off of the MHV patient portal. This can include difficulty filling out SMMRT and saving SMMRT. This can also include sociotechnical obstacles barriers to completing SMMRT (eg, patient’s health).	“I had a problem [with SMMRT] because I couldn’t save the stuff anyway... I went over each of the prescriptions and it asked what my dosage was, was I taking it, and stuff like that. When we did [complete] that, the ‘submit to save’ [option] never worked”
Ease of use of SMMRT	Positive comments about using SMMRT PDF once downloaded. This includes ability to use drop down boxes, ability to use free text boxes, text size, and readability of the PDF.	From a caregiver: “It was quite easy. It was very easy, and I think the form was pretty good to verify [the patient’s] medication.”
Training and technical support	Comments about perceptions of the education, instructional materials, and technical support offered to participant with regards to SMMRT study process. This includes in-person training in the hospital, written “help guide” sent home with participant, and phone calls with study team for technical help.	“It was fairly easy. [The RA] basically explained pretty clearly and [in] very easy terms what the task at hand was, and pretty much once I logged in, that was very clear. She also gave me some handouts. It was pretty easy for me to comprehend and follow.”
Effectiveness of SMMRT	Benefits of using the SMMRT PDF. This can include comments about medication clarifications that occurred as a result of using SMMRT PDF, other secondary benefits experienced by the participant (eg, increased MHV use), and positive overall thoughts and feelings about the study. This can also include comments about follow up from study pharmacists.	“…even though I thought there was no way I could do that [ie, make an error], I had misinterpreted the one of the instructions on my medications and your [SMMRT] program caught it.” “The SMMRT study itself I think is kind of long overdue… this could become a regular practice or a regular part of MyHealtheVet or somehow incorporated in the whole experience, I think it will be very helpful for people. I found it was for me.”
Facilitated SMMRT form completion	Comments about completing SMMRT over the phone with extensive assistance from a pharmacist or study staff (eg, pharmacist completes SMMRT during the call based on conversation with the patient). Exclusion: This does not include comments relating to technical support completing SMMRT or comments relating to follow-up calls from pharmacist to reconcile identified medication discrepancies.	“I had already had [SMMRT completed] on my computer anyway so it was really easy for me to translate it to him that way [when he called]”
Medication pictures on SMMRT	Comments about the value (good or bad) of the medication pictures included on the SMMRT PDF.	“Pictures of medications? No, I don’t remember any pictures.” “I think it would be good if there were pictures on every one of them. Only because when [patients] have their pill box, and if they have the picture of it, even though some are the same color and shape, they might have an idea of which one is which if it’s all in a pill box.”
Future directions	Comments about future development and use of SMMRT. This includes whether the participant would recommend it to other veterans and whether addition training tools, such as YouTube would be beneficial. This can also include suggestions of changes to the design and use of SMMRT PDF.	“Well, I guess if they have computer knowledge… [and] they’re comfortable using the computer, I don’t see why anybody would object to doing this.”

^a^SMMRT: Secure Messaging for Medication Reconciliation Tool.

## Results

### Study Sample

There were 20 interviewees, who were all male, and the majority were white (13/20, 65%). They had a mean age of 62.5 (SD 9.5) years and had completed a mean of 13.8 (SD 2.4) years of education. Demographics of the sample for these interviews were consistent with the demographics of the overall study sample. Participant characteristics are displayed in Table 2. Most (16/20, 80%) patients had registered for a MyHealtheVet account prior to enrollment in the trial, and 15 (15/20, 75%) self-reported previous secure messaging use. Among those who reported regular daily computer use (16/20, 80% of total sample), most also reported prior secure messaging experience (14/16, 88%). One participant reported that a caregiver used secure messaging on his behalf and the patient himself had no computer experience. Thus, we recruited the caregiver to use SMMRT in collaboration with the patient, and both participated in the interview.

Interviews were conducted at an average of 34.8 (SD 19) days following the participants’ completion and submission of SMMRT. Self-reported viewing of SMMRT was analyzed and used to categorize participants into viewed (n=17) and did not view (n=3) SMMRT on a home computer. We also assigned SMMRT return status based on data collected in the larger clinical trial: returned SMMRT via secure messaging (n=9), completed SMMRT via telephone with a clinical pharmacist (n=10), and did not complete SMMRT (n=1).

During the qualitative analysis process, we identified findings related to 7 themes, which included Training and Technical Support, Medication Pictures, Technology-related Barriers, Pharmacist-Facilitated SMMRT Completion, SMMRT Completion, Perceived Effectiveness of SMMRT, and Future Development.

**Table 2:** Characteristics of patient participants (n=20).

Characteristics		Results
Age (years), mean (SD)		62.5 (9.5)
Male, n (%)		20 (100)
**Race,** **n (%)**		
	White	13 (65)
	Other	7 (35)
**Education level, n (%)**		
	Completed grades 8-11	2 (10)
	High school/general educational development (GED)	5 (25)
	Some college	7 (35)
	College graduate or higher	6 (30)
**Employment status, n (%)**		
	Full time	6 (30)
	Part time	2 (10)
	Retired	6 (30)
	Unemployed	1 (5)
	Disability	5 (25)
**Self-reported computer use, n (%)**		
	Never^a^	1 (5)
	A few times	4 (20)
	Regularly	12 (60)
	Expert	3 (15)
Prior patient portal experience^b^, n (%)		16 (80)
Prior secure messaging experience, n (%)		15 (75)
SMMRT^c^ viewing status: viewed SMMRT on home computer (self-report), n (%)		17 (85)
**SMMRT completion status, n (%)**		
	Completed and returned via secure messaging	9 (45)
	Completed with pharmacist via telephone	10 (50)
	Not completed or returned	1 (5)
Number of active medications in the EHR^d^, mean (SD)		15 (6)
Days between SMMRT completion and interview, mean (SD)		35 (19)

^a^One participant reported relying on the assistance of a caregiver when completing SMMRT and while using the patient portal, MyHealtheVet.

^b^The portal was MyHealtheVet.

^c^SMMRT: Secure Messaging for Medication Reconciliation Tool.

^d^EHR: electronic health record.

### Training and Technical Support

Participants with no previous secure messaging experience (n=5) reported the training session was very valuable, with 1 participant reporting “Without it, I wouldn’t have been able to get the job done.” A user that had previous MyHealtheVet experience endorsed the benefits of the training, saying the “[RA] made some small suggestions for me so I could understand it a little better. It was great and beneficial as a refresher course.”

### Medication Pictures

Although SMMRT contained sample pictures of each medication listed, few participants reported favorable opinions relating to this detail, with many not recalling the medication pictures on SMMRT. Importantly, no participants reported issues with incorrect picture images (eg, a picture of a generic form of the medication vs a picture of the name-brand medication).

### Technology-Related Barriers to Returning SMMRT Electronically via Secure Messaging

Of the 20 participants, 11 (55%) did not return SMMRT via secure messaging. Barriers were primarily related to technology and included 3 main subthemes: (1) difficulties with using a PDF (eg, saving the completed SMMRT to the computer prior to upload), (2) patient portal access issues (eg, difficulty logging into the portal and problems attaching SMMRT to the secure message), and (3) internet bandwidth issues. Participants also described difficulty with downloading SMMRT from the patient portal. For instance, “it wouldn’t let me download all the [SMMRT] pages.” Some of these issues were also raised by participants who were able to overcome these barriers to return SMMRT via secure messaging. In some situations, participants contacted the study team for technical support, while other barriers were resolved by the participant using the user guide provided during study training. A limited number of participants reported contacting the study team for help troubleshooting issues relating to downloading SMMRT and sending SMMRT back to the appropriate person.

The level of internet connectivity required for use of the patient portal and SMMRT was also discussed by participants. Participants reported difficulty with downloading and sending SMMRT due to limited internet connectivity, with 1 participant reporting, “I live in the mountains with no internet hardly. I have to get [internet] through a satellite,” which then led to decreased internet bandwidth speed. This highlights the access issues some rural veterans may experience when trying to access the patient portal to complete SMMRT.

### Pharmacist-Facilitated SMMRT Completion

Due to difficulty returning SMMRT via secure messaging, 10 participants (10/20, 50%) reported completing SMMRT over the phone with a clinical pharmacist. Participants reported this collaboration with the pharmacist to be smooth and efficient, with most participants reporting it took less than 15 minutes to complete SMMRT over the phone. Patients who completed SMMRT over the phone with assistance from a pharmacist (n=10) had a mean age of 63.7 (SD 9.5) years compared with a mean age of 60.4 (SD 11.7) years for patients who completed SMMRT via secure messaging (n=9). Of the patients completing SMMRT with the pharmacist, 6 reported their computer expertise as a “regular” or “expert” user, with the other 4 patients reporting limited computer experience. In contrast, all 9 patients who returned SMMRT via secure messaging reported their computer expertise as a “regular” or “expert” user.

### SMMRT Completion

Of those who viewed SMMRT in the patient portal, most participants reported that SMMRT was easy to navigate, with participants reporting “it was pretty easy to comprehend and follow” and “everything was great and very clear, very straightforward.” Participants who returned SMMRT via secure messaging reported that the directions were clear, allowing for a seamless completion of SMMRT and emphasized the benefit of completing SMMRT in the comfort of their own home with their home medication list, without the stress of being in a medical setting. Of participants who were able to complete SMMRT (n=9), the mean self-reported time to complete SMMRT per participant was 13.6 (SD 3.23) minutes. Participants expressed that SMMRT was timesaving in comparison with the usual medication reconciliation process they commonly experience postdischarge.

### Perceived Effectiveness of SMMRT

The perceived effectiveness of SMMRT by the participants was discussed in the context of 2 key categories: (1) reconciling medications that were previously discontinued by a medical provider but were never removed from the patient’s “active” medication list in the EHR and (2) clarifying medication dosages or frequencies. Overall, the majority of patients who completed SMMRT via secure messaging or in collaboration with the clinical pharmacist reported that the use of SMMRT helped remove at least one medication from the patient’s health record that was erroneously listed as “active” but had been previously discontinued by a medical provider. Additionally, participants reported that SMMRT helped clarify an issue relating to medication directions or dosages. One participant stated, “[The pharmacist] deleted what was necessary, and by the time we had the [trial follow up call], [my medication list] was clean.” Participants reported feeling “more in tune” to their medications after completing SMMRT.

### Future Development

Nearly all patients who viewed SMMRT stated they would recommend it to other patients transitioning from hospital to home. Participants suggested adaptations, such as providing delivery via a smartphone app, online video tutorials, and increased availability of on-demand help via the internet. Participants explained that these resources could better assist individuals who have limited computer knowledge and thus advance the ease of using the patient portal, secure messaging, and therefore SMMRT. Overall, participants endorsed the need for a tool like SMMRT, stating that this type of medication management technology is “long overdue.”

## Discussion

### Principal Findings

In this study, we examined the perspectives and preferences of patients who used SMMRT to conduct medication reconciliation from home after a recent hospitalization. We captured patients’ perspectives on the effectiveness of SMMRT and its ability to help uncover medication discrepancies, the visual display of SMMRT, the barriers faced when completing SMMRT, and the potential future use of SMMRT in routine patient care. To our knowledge, this is one of the first studies to specifically assess patients’ user experiences with secure messaging for medication reconciliation after hospital discharge.

Most patients completed SMMRT via secure messaging or in collaboration with the clinical pharmacist. More than one-half of patients in our sample identified a medication discrepancy via SMMRT, highlighting its clinical effectiveness. If patients perceive that digital technologies are effective, it often increases the likelihood that the technology will be utilized [31]. This may have promoted patients’ overall favorable opinions of SMMRT, despite the barriers experienced when returning SMMRT via secure messaging. Recent literature reports that patients can identify medication discrepancies in their own personal health record with comparable accuracy to a pharmacy technician, supporting the assertion that patients can be closely involved in the medication reconciliation process [32,33], as they were in the SMMRT intervention. SMMRT has the potential to reduce the risk of ADEs and rehospitalizations by providing a mechanism for patients to help identify medication discrepancies.

Our study revealed insights on the visual display of SMMRT, which can inform future medication reconciliation technologies for patients. For example, we found that the medication pictures did not hold significant value for the patients. This study finding is in contrast with prior usability research from our group during SMMRT development that indicated strong support for including medication pictures [23,34]. Additionally, interviewed patients did not indicate any confusion regarding the presence of the pictures, even though this was reported to be an issue during the initial usability testing of SMMRT [23]. Together, these findings indicate that the medication pictures did not benefit nor harm patients’ user experience. Thus, our findings indicate that the resources needed to include accurate medication pictures on SMMRT may exceed their value to patients.

Despite our overall positive findings, patients’ ability to return SMMRT electronically was impeded by many barriers, with patient portal–related usability problems among the most prominent obstacle. This is likely due to the portal’s file uploading process, which involves multiple critical steps. In addition, barriers related to geographic infrastructure, such as patients with poor or no internet connection, also created a disadvantage for patients living in more rural or remote areas. Alternative options for medication reconciliation during transitions of care may be needed for that population. One way to reduce some barriers is by adapting SMMRT for use on a smartphone. This adaptation may further increase patient uptake given the role that smartphones play in many day-to-day activities, possibly leading to fewer barriers faced. Patient portal use may increase with the perceptions that increased use may result in increased long-term independence [35], possibly explaining older patients’ willingness to learn, troubleshoot, and use this new tool despite the difficulties experienced. Improving overall usability of patient portals and associated secure messaging technologies may increase the adoption of SMMRT and medication-related tools for patients of all ages and technology-related abilities.

Overall, patients endorsed SMMRT to be a valuable medication reconciliation tool and indicated they would use SMMRT again. Similar to our findings, prior research has found secure messaging to be beneficial and useful for regular communication [17,36]. Nevertheless, adoption of medication reconciliation technologies remains low [37]. When reviewing the literature, we found studies that examined several other types of medication reconciliation tools [12,34,38], such as clinician-facing medication reconciliation tools for use in the hospital setting [9] or kiosks for patients to review and comment on their medications prior to their clinical appointment [9,34]. Other studies evaluated secure messaging, primarily for purposes other than medication reconciliation [20,21]. Thus, it is difficult to contrast our findings with others due to the novelty of our research and lack of published research on medication reconciliation with a similar clinical process (postdischarge), technology (secure messaging), and study setting (patient’s home use). Importantly, our findings provide evidence that patients are willing to engage with medication reconciliation technologies in the home setting, after hospitalization, and find them useful for medication management.

### Limitations

This study was conducted with 1 patient portal, which although used for veteran patients across the United States, may differ in some ways from other commonly used patient portals. In addition, although both men and women were eligible to participate in the interviews, only men were interviewed, reflecting much of the patient population of the VA health care system. The high mean age of the patients in this sample may have influenced the findings. Patient age may influence patient portal use, with research showing older patients experience greater barriers [39] to use than younger patients. We used sequential sampling due to project constraints, but the use of random sampling would have been a stronger approach.

Although the follow-up interviews to the larger clinical trial included aspects of usability, they were not specifically focused on usability; thus, a usability framework was not used. Incorporating a usability framework to the interview guide or analysis process could provide increased value. The use of a usability questionnaire as part of the interview could have also yielded additional insights.

We also only examined the perspectives of patients, not clinicians, who are also instrumental in medication reconciliation processes. Lastly, qualitative interviews were conducted up to 2 months after patients completed SMMRT, which may have affected recall; however, patients had the option to view SMMRT while completing the interview. Future research is warranted to further enhance the design and usability of SMMRT, secure messaging systems, and patient portals.

### Conclusion

Our findings offer insight into the usability of an at-home medication reconciliation tool—SMMRT. Overall, this tool was viewed as a positive and valuable experience by most patients. Patients perceived SMMRT to be an effective mechanism to conduct medication reconciliation after a recent hospitalization, and nearly all patients stated they would recommend SMMRT to other patients. Importantly, for more than one-half of patients in our study, the use of SMMRT uncovered at least one medication discrepancy. Digital health tools such as SMMRT offer increased ownership for patients over their own personal health information and may lead to greater overall health compliance, highlighting the need for continued development of SMMRT. If widely implemented, SMMRT has the potential to improve medication safety on a much larger scale. Nonetheless, we identified several barriers that should be addressed, with barriers relating to the patient portal being the most prominent. Additional efforts are warranted to improve the usability of SMMRT and secure messaging platforms for patients.

Our results are expected to be valuable to health care organizations, software developers of patient portals and secure messaging platforms, and patients themselves. Implementing SMMRT into routine clinical practice could allow for greater patient involvement and enhanced medication safety during the vulnerable transition from hospital to home.

## Appendix

Multimedia Appendix 1 Semistructured interview guide.

## Abbreviations

ADE: adverse drug event
EHR: electronic health record
HSR&D: Health Services Research and Development
RA: research assistant
SMMRT: Secure Messaging for Medication Reconciliation Tool
SRQR: Standards for Reporting Qualitative Research
VA: Veterans Affairs
