# Supplementary material for: The Perceived Effectiveness of Secure Messaging for Medication Reconciliation During Transitions of Care: Semistructured Interviews With Patients
Source: JMIR Hum Factors. 2022 Aug 3;9(3):e36652. doi: 10.2196/36652 (PMC9386577; doi:10.2196/36652)
Supplement: Multimedia Appendix 1 [file humanfactors_v9i3e36652_app1.docx]

**Semi-Structured Interview Guide for Interviewing Participants**

Today, I would like to talk with you about your experiences as a participant in our research study, which we call SMMRT Trial. As you may recall, the study randomly allocated you to receive the intervention that provided you with training in the use of My HealtheVet and the use of the Secure Messaging for Medication Reconciliation Tool.

1. What were your overall impressions of participating in this study?
2. Thinking back to when you were in the hospital and agreed to participate, how did you think that process went?
   1. Clarify if necessary: Did it go smoothly? Did it take longer than you think it should have?
3. What do you remember about the training for My HealtheVet?
4. What parts of the training, if any, did you find helpful?
5. What parts of the training, if any, did you think were unhelpful or even counter-productive?
6. What were your general impressions of the Secure Messaging for Medication Reconciliation Tool, or SMMRT Tool?
   1. Clarify: Was it easy or difficult to use. Why?
7. What elements of SMMRT were helpful?
8. What elements of SMMRT tool were not helpful, or even counter-productive?
9. What can you tell us about your experience in exchanging messages with the study clinical pharmacist to review your medications?
10. Let me shift gears for a moment. We are interested to know how your involvement in this research study may have affected your interactions with your regular health care team. Do you have any initial thoughts or comments before I ask some specific questions?

Those are all of our questions. Is there anything else about your participation in the study that you would like to tell us, or do you have any questions?
